# Protocol for a quasi-experimental analysis: using retail sales data to evaluate impacts of the high fat, sugar and salt (HFSS) product placement restrictions legislation in England

**DOI:** 10.1136/bmjph-2024-002065

**Published:** 2025-12-25

**Authors:** Victoria L Jenneson, Francesca Pontin, Emily Ennis, Alison Fildes, Alexandra M Johnstone, Michelle A Morris, on behalf of the DIO Food team, Alexandra Johnstone

**Affiliations:** 1 School of Food Science and Nutrition, University of Leeds, Leeds, UK; 2 Leeds Institute for Data Analytics, University of Leeds, Leeds, England, UK; 3 School of Geography, University of Leeds, Leeds, UK; 4 School of Psychology, University of Leeds, Leeds, UK; 5 The Rowett Institute, University of Aberdeen, Aberdeen, Scotland, UK

**Keywords:** Public Health, Nutrition Assessment, Nutritive Value

## Abstract

**Introduction:**

From 1 October 2022, new legislation in England restricts the placement of some food and drink products high in fat, sugar and salt (HFSS) in stores with a sales floor larger than 185.8 m^2^ (2000 sq ft). ‘Less healthy’ products in 13 categories, which fail the UK Nutrient Profiling Model, cannot be placed at store entrances, ends of aisles, at checkouts or their online equivalents. Reducing the prominence of ‘less healthy’ foods should decrease impulse purchases and excess calorie intake. Stores in Scotland, Wales and Northern Ireland are exempt, but the UK Devolved Nations are considering similar rules.

**Methods and analysis:**

Daily store-level sales and product data from multiple UK retailers will be used to evaluate the legislation’s impacts in relation to HFSS sales, retailer product portfolios and equitability across different areas in England. Food and drink sales data from 18 months pre-policy and 12 months post-policy implementation will be gained for a sample of stores. Controlled interrupted time series modelling will be used to estimate policy effects, with stores from Scotland and Wales acting as controls. Online sales are excluded.

Stores from study partner retailers were identified using Geolytix retail points data. Selected stores were defined as mid-sized or larger (>280 m) by Geolytix, to ensure legislation eligibility. 160 stores in England (intervention sample) and 50 stores in Scotland/Wales (control sample) were selected for each partner retailer. For each retailer, we sampled equal intervention store numbers (n=16) across each tenth of the Priority Places for Food Index (PPFI). Weighting was used to ensure maximum sample variability by store size and area demographic characteristics (urban/rural status) within each PPFI tenth.

**Ethics and dissemination:**

This study has received approval from the Business, Environment and Social Sciences ethical review board at the University of Leeds (AREA 21–063).

Findings at the retailer and cross-retailer levels will be published in academic journal articles as well as industry-facing reports coproduced by Institute of Grocery Distribution. Through meetings and workshops, we will disseminate results to inform future business practice and policymaking across the UK Devolved Nations.

**Registration:**

Protocol was first published on Open Science Framework https://osf.io/jp9eh.

**WHAT IS ALREADY KNOWN ON THIS TOPIC**

- Prominent placement of ‘unhealthy’ products in store can lead to overconsumption. High in fat, sugar and salt (HFSS) legislation in England now restricts product placement to reduce impulse purchasing but evidence of effectiveness in practice is in its infancy.

**WHAT THIS STUDY ADDS**

- This study is the first cross-retailer independent analysis on the effectiveness of England’s HFSS legislation. We present a protocol outlining plans for robust policy evaluation using controlled interrupted time series.

**HOW THIS STUDY MIGHT AFFECT RESEARCH, PRACTICE OR POLICY**

- Findings from this study will inform the roll-out of future HFSS legislation in the UK devolved nations and beyond. It will also inform how collaborative working can unlock the power of repurposed sales data for cross-sector public health policy insight.

## Introduction

The presence of less healthy products in prime store locations can increase sales by 20%–61%^1 2^ and lead to negative dietary outcomes.^3 4^ For example, confectionery items are frequently purchased on impulse^5^ and over-consumed in the UK diet.^6^ A 25% population-level reduction in products high in salt, sugar or salt (HFSS) is required to meet public health guidance.^7^ Experimental evidence indicates that removing unhealthy products from prominent store locations can reduce sales and potentially improve diets.^8^,^10^

On 1 October 2022, new legislation (henceforth the ‘HFSS legislation’ or ‘the legislation’) was introduced for England, restricting the placement of some food and non-alcoholic beverage products in store and online.^11^ By reducing the prominence of unhealthy foods, the legislation aims to reduce impulse purchasing and subsequent over-consumption. In scope products can no longer be placed in prime store locations, including ends of aisles, near store entrances and the checkouts. In scope categories (n=13: soft drinks with added sugar, crisps and savoury snacks, breakfast cereals, confectionery, ice creams and ice lollies, cakes, biscuits, breakfast bakery, desserts and puddings, yoghurt, pizza, potato-based products and ready meals) represent commonly consumed processed foods, which contribute significantly to excess calorie intake.^11^ Prepackaged products, which are classed as ‘less healthy’ by the UK’s 2004/5 Nutrient Profiling Model (NPM),^12^ are considered to be high in saturated fat, sugar and/or salt (HFSS) and in scope for the legislation.

Prior to the legislation, an estimated 43% of prime store locations in the UK contained HFSS products^13^ with HFSS products making up 62% of total food sales in Great Britain. Government impact assessment modelling suggested that restrictions to HFSS product placement could bring significant societal cost benefits, with a net gain in the region of £68 m,^14^ including savings to the National Health Service (NHS) and social care and accounting for estimated enforcement costs.

Responsibility for implementation and adherence is placed on retailers, with sanctions threatened for non-compliance. The legislation is expected to act predominantly through the reduced prominence of HFSS items in retail settings, but knock-on effects such as product reformulation are also anticipated. Indeed, surveys with food industry actors (conducted by Institute of Grocery Distribution (IGD)) point to a substantial focus on reformulation, incentivised by the opportunity to achieve an NPM score which exempts products from product placement restrictions.^15^ In addition to reformulation, there are significant costs associated with implementation, in store and online, with initial expected costs to retailers in the region of £13 000 per store for smaller shops and £100 000 for larger shops^16^ and estimated total retailer implementation costs upwards of £47 m, with a following £17 m for ongoing product assessment, alongside £2947 m profit losses.^14^ While there is some short-term evidence from Co-op stores that, despite changes to customer purchases, the legislation had no impact on profitability,^17^ high stakes for the legislation warrant a thorough evaluation of its impacts.

More than 1 year since the legislation was introduced in England, the UK’s Devolved Nations of Scotland and Wales are considering introducing similar policies. However, at the time of writing, evidence of the policy’s effectiveness is in its infancy, with the majority of evidence from industry sources. Market research companies Kantar and Neilsen IQ (NIQ) suggest positive shifts towards purchases of healthier options. According to Kantar consumer panel data, overall sales of ‘healthier’ non-HFSS options rose by 16% in the first 12 weeks of the legislation, compared with a more modest 6% rise among HFSS product counterparts.^18^ Share of the food and drink market for HFSS products has also reportedly declined from prelegislation levels (16%, or £18 billion) while shares for non-HFSS products have increased by £34.4 million.^18^ However, it is unclear what this means in real terms (eg, product volumes and calories purchased) for population dietary habits. Indeed, legislation effectiveness may vary at the category level. Industry reports cite lower policy success in ‘ambient’ products (category not well defined)^5^ and greater successes in savoury product reformulation (with an average 0.5 point decline in NPM score)^18^ contributing to growing sales of ‘healthier crisps’ (up >50%)^19^ and declining HFSS sales in both pizzas and crisps.^5^

Change among sweet products may be more difficult to achieve. Evidence from Kantar suggests that contrary to the policy’s objectives, the average NPM score for sweet confectionery *increased* by 0.4 points (becoming less healthy),^18^ coupled with relatively modest growth in non-HFSS confectionery, equating to just 6% of the confectionery market.^5^ Indeed, findings for confectionery are somewhat contradictory. While Kantar reports that total sales of confectionery (particularly chocolates) have fallen 3.4% in the week ending 3 June 2023,^5^ market analysts Reapp reported an additional £50 million rise in chocolate sales and 100 000 unit increase in sweet sales over the year postlegislation.^20^ Such contradictions highlight an important concern for bias and selective reporting among industry-led analyses. A lack of methodological reporting in the current evidence-based adds weight to the need for in-depth robust independent analysis of the HFSS legislation. Furthermore, there have been no reports on unintended consequences in out-of-scope categories, such as alcohol.

Adequate enforcement is required to incentivise ongoing industry efforts, yet comes with challenges. With Trading Standards enforcement budgets proportionally slim, estimated to be between £35 000^5^ and £179 000^21^ split between 344 local authorities in the first year, enforcement efforts are likely to be limited. Currently, little is known about enforcement actions^5^ and where breaches have been identified, no improvement notices had been issued at the time of reporting.^22^ Greater understanding of enforcement monitoring is, therefore, important but outside the scope of this research.

The study aims to understand the impact of the HFSS legislation on product offerings, store-level sales of HFSS products and equitability across different communities. Secondary data from multiple major UK supermarkets will provide sector-level insights. To our knowledge, this protocol outlines the first multiretailer independent analysis of the HFSS legislation and uniquely explores the equitability of impacts across different socioeconomic groups. Our analyses will focus on insights from in-store settings; thus, we exclude sales made on online grocery platforms. Retailer interviews and surveys, as well as customer surveys (described elsewhere),^23^ will add context to the patterns observed. These timely insights could feed into development of similar policies for the UK devolved nations (and beyond), and ahead of upcoming legislation to restrict volume-based price promotions for HFSS products, due to be introduced in England in October 2025.

## Methods and analysis

Data sharing partnerships were formed with four major UK retailers in 2024; ASDA, Morrisons, Sainsbury’s and Tesco, who collectively represent 65% of supermarket sales^24^ in England (as of April 2024). Store-level sales data will enable examination of changes to HFSS and non-HFSS product sales since the legislation’s introduction. This protocol was developed in spring 2024, with analysis of the sales data to answer the research questions set out here, to begin in summer 2024. The anticipated project end is summer 2026.

### Definition of the intervention

Since 1 October 2022^25^ in England, products in scope for the legislation (referred to as ‘specified foods’) cannot be placed within 2 m of store checkouts and designated queueing spaces, at the ends of aisles or within 50 cm thereof, and within a given distance from the store entrance (prohibited distance calculated based on relevant store floor area), and online equivalent spaces. Restrictions apply to stores with a minimum relevant floor area of 2000 sqft (185.8 m^2^) (excluding non-sellable floor space, eg, stockrooms, staff rooms and toilets), which belong to ‘qualifying businesses’, medium or large businesses employing 50 or more staff. Specialist retailers which mainly sell a single category of food (eg, chocolatiers) are exempt from the legislation. Online sales are excluded from the scope of this study.

**Figure 1:**
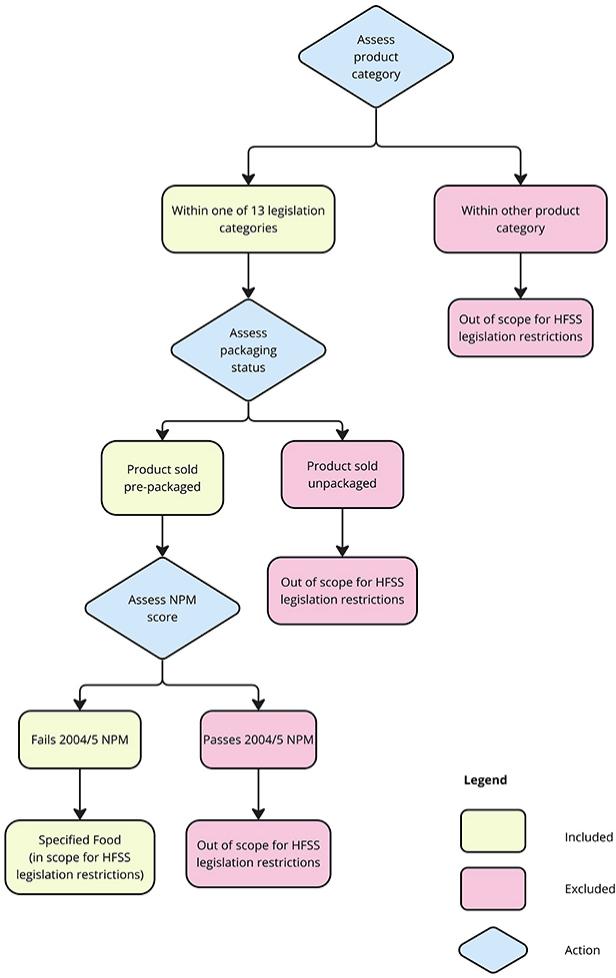
Decision tree outlining the identification of ‘Specified Foods’ eligible for placement-based restrictions under the HFSS legislation. HFSS, high in saturated fat, sugar and/or salt; NPM, Nutrient Profiling Model.

Specified foods (depicted in figure 1) are prepackaged products within 13 legislation product categories: soft drinks with added sugar, crisps and savoury snacks, breakfast cereals, confectionery, ice creams and ice lollies, cakes, biscuits, breakfast bakery, desserts and puddings, yoghurt, pizza, potato-based products, and ready meals. Loose items such as in-store bakery products are exempt from the legislation. In scope products which fail the UK 2004/5 NPM^12^ are eligible for restrictions.

The UK 2004/2005 NPM calculates an overall score based on nutrient quantities per 100 g of product, whereby a higher overall score indicates a less healthy product. A-points are allocated for health-harming nutritive components: energy, saturated fat, sugars and sodium and C-points are allocated for health-promoting nutritive components: fibre, protein and fruit, vegetable and nut percentage by weight. C-points are subtracted from A-points to give an overall NPM score. Food products with a score of 4 or greater, and non-alcoholic beverages with a score of 1 or greater are considered ‘less healthy’ or to have ‘failed’ the NPM.

### Store sample selection

Store selection has been conducted to include an equal number of stores from each partner retailer, N=160/retailer in the intervention group (stores in England) and N=50 stores/retailer in the control group (stores in Scotland and Wales, where the legislation was not implemented). Eligible stores were identified from the 2023 Geolytix Retail Points (V.28) dataset, which contains store locations for all major retailers in the UK.^26^ Next, an a-priori sampling frame was used to select included stores from the eligible store pool.

#### Identifying eligible stores

**Figure 2:**
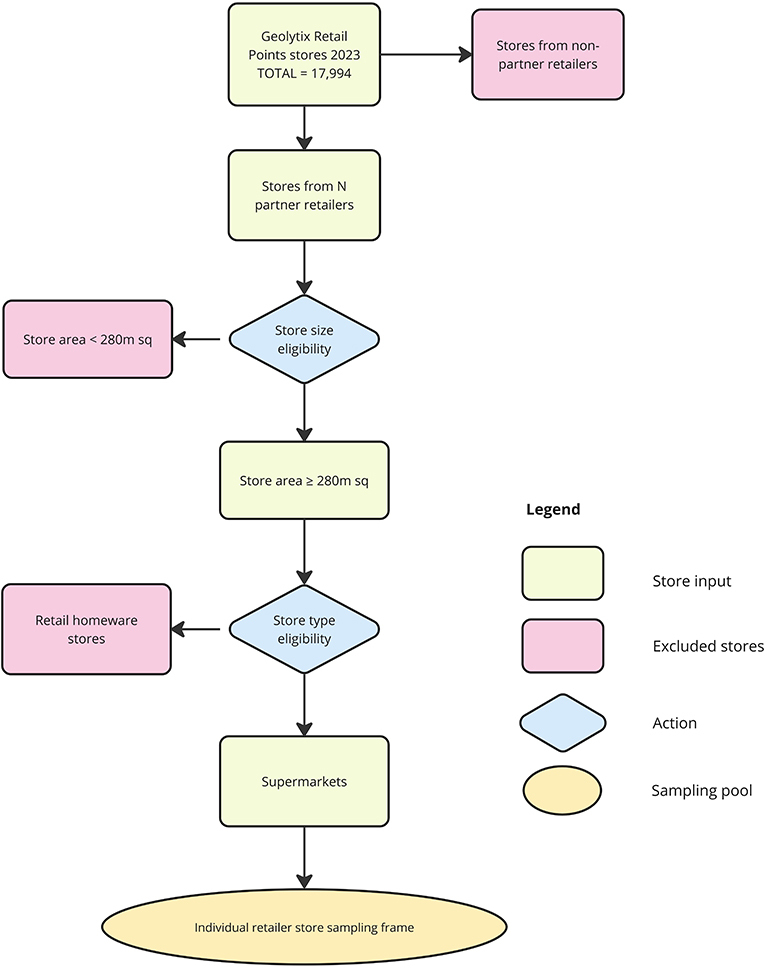
Flowchart depicting the identification of eligible stores to be passed into the sampling frame. The purpose was to select stores eligible for the legislation in England and equivalent counterparts in devolved nations of Scotland and Wales.

The eligible store pool (figure 2) was designed to include all stores from our partner retailers in England, Scotland and Wales, which sell foods and beverages and meet legislation store size requirements, using features in the Geolytix database. As Geolytix store size bands do not align exactly with legislation store size criteria (< 280 m^2^; 280 <1400 m^2^; 1,400<2800 m^2^ and >2800 m^2^) (Geolytix, 2023) and represent the whole store footprint rather than relevant sellable floor space, small stores in the <280 m^2^ size band were excluded, increasing the likelihood of including in scope stores only. Actual store sizes and appropriateness of store selection under HFSS rules were verified by retailers prior to analysis. Retail homeware stores (identified using the Geolytix ‘fascia’ variable) were also excluded.

The eligible store pool over-indexed on larger stores and stores in urban locations. To correct for this, our a-priori sampling frame employed a maximum variation sampling approach to maximise the breadth of area and store characteristics in the final sample. Oversampling techniques (based on store size and urban/rural area characteristics) were applied to maximise the probability of selecting edge case scenarios (ie, smaller stores in rural locations).

#### Intervention group stores

**Figure 3:**
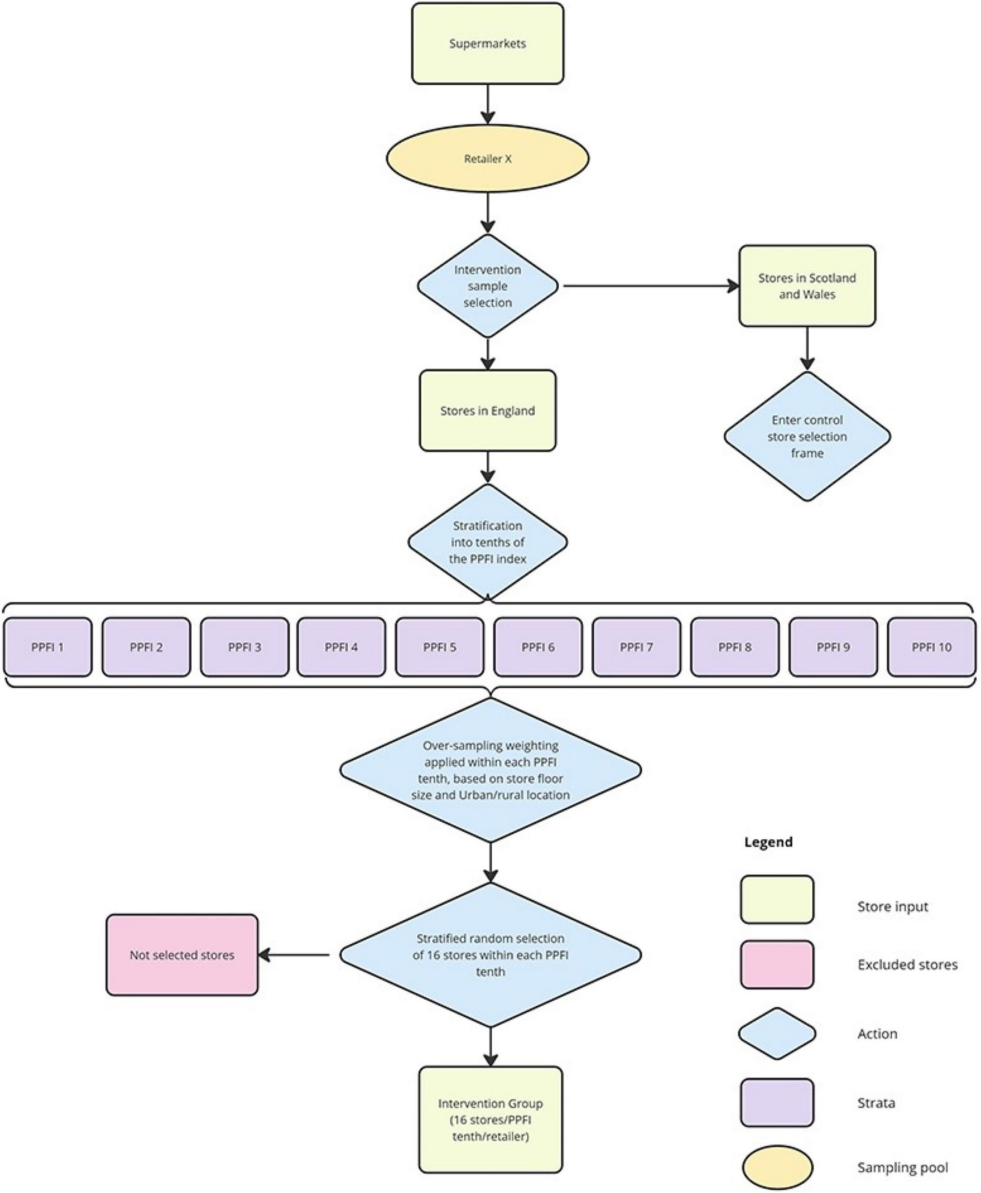
Retailer-level intervention store sampling frrame. The purpose was to select an equal sample of stores per retailer (n=160), with an equal distribution across tenths of the PPFI per retailer (n=16). Oversampling by floor size and rural/urban location are applied to maximise sample diversity. PPFI, Priority Places for Food Index.

The process flow diagram in figure 3 outlines the selection of intervention stores from the eligible store pool. Stratifying by retailer, 160 intervention stores were selected, representing 16 stores from each tenth of the Priority Places for Food Index (PPFI)^27^ (at the Lower Super Output Area level for England and Wales, and Data Zone level for Scotland). Neighbourhoods in decile 1 of the PPFI are considered in the 10% highest priority for support accessing food, in terms of access and financial barriers to food.

Within each PPFI decile strata, weightings were applied based on the number of stores with each unique store size band (1=small (280–1400 m^2^), 2=medium (1400–2800 m^2^) and 3=large (>2800 m^2^)) and urban–rural area 2011 (ONS, 2016) classification combination. Weightings were calculated as the complement of selection (1 minus the original probability of selection), which up-weights stores with under-represented size-urban/rural combinations (increasing their probability of selection) and down-weights over-represented size-urban/rural combinations. For example, in a hypothetical scenario where one retailer has 25 stores in the highest priority tenth of PPFI (decile 1), from which we want to select 16 of these stores. Only 3 of the 25 stores have the store characteristic combination of small store in a rural area, meaning this combination of characteristics is under-represented and less likely to be selected into the final sample. To improve the probability of selecting stores with these characteristics, the original probability of selection is calculated 3/25=0.12, then deducted from 1 to give a new probability of selection 1–0.12=0.88. The new probability of selection is then used as a weight in the Python sampling algorithm (pandas.DataFrame.sample, V.1.4.0), which treats the probability of selecting a small store in a rural area as if 22 of the 25 stores have this characteristic (more likely to be chosen).

#### Control group stores

Control stores were selected for Scotland and Wales to account for history bias (other interventions or events that occurred at the same time as the intervention) affecting trends in purchasing behaviour independent of the HFSS legislation. Stores in Scotland and Wales are likely to have been subjected to the same supply chain issues, changes in population purchase habits as a result of the COVID-19 pandemic and effects of the cost of living crisis as stores in England, thus acting as reasonable controls.

**Figure 4:**
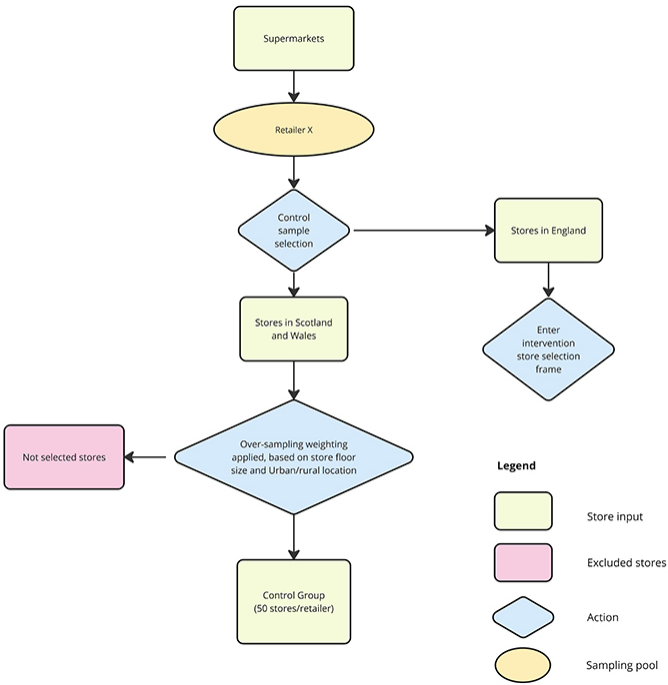
Retailer-level control store sampling frame. The purpose was to select an equal sample of control stores per retailer (n=50). Due to low numbers, stratification by PPFI tenths not applied. Oversampling by floor size and rural/urban location are applied to maximise sample diversity. PPFI, Priority Places for Food Index.

Due to small store numbers in Scotland and Wales, it was not possible to select an equal number of control stores within each tenth of the PPFI. Therefore, stratification by PPFI decile was not performed prior to control store selection. To maximise control group size (while maintaining equality in numbers across each retailer), we request data for 50 control stores in the devolved nations of Scotland and Wales per retailer. As per the intervention store sampling frame, reweighting based on the probability of selecting stores with each size-urban/rural characteristic pairing was applied, though at the whole eligible pool level, rather than within PPFI strata. An overview of the control store sampling frame can be found in the flow diagram in figure 4.

### Sample size calculation

As the unit of analysis is supermarket stores, rather than individuals, our sample size strategy maximises the number of included stores while ensuring equal data sharing requirements for each retailer. Selecting an equal number of stores from each PPFI decile maximises statistical power to examine differences in outcomes across stores from different tenths of the PPFI.

There are no accepted methods for calculating sample sizes required for controlled interrupted time-series methods, nor performing power calculations.^28^ However, it is generally accepted that more timepoints and a greater number of observations per timepoint will provide greater statistical power, while higher autocorrelation decreases statistical power. For Autoregressive Integrated Moving Average (ARIMA) models, which underpin the interrupted time-series methodology, a minimum of 50 timepoints is an accepted rule of thumb.^28^

Our design maximises the number of timepoints by collecting daily store-level sales, providing a total of 913 observation days per retailer (1 April 2021—30 September 2023), including 548 days (18 months) in the preintervention period and 365 days (12 months) in the postintervention period. With 160 intervention and 50 control stores, this equates to 146 080 store-day pairs per retailer for the intervention group and 45 650 store-day pairs per retailer for the control group. We anticipate this sample size to be sufficient to detect a 1 percentage point change in sales of HFSS products at the 95% level.

### Outcomes

Analysis will address four research questions, underpinned by subquestions (primary research question first). Outputs will be presented at the retailer level with the potential for aggregation across retailers. Protocol was first published on Open Science Framework https://osf.io/jp9eh.

#### Q1—What happened to HFSS product sales after the introduction of the policy?

Controlled interrupted time-series will reveal whether sales of HFSS products reduced following introduction of the legislation using percentage point change in total HFSS sales (ie, products which fail the NPM, regardless of ‘Specified Foods’ status) as a proportion of total sales (by weight) as the primary outcome. HFSS sales will be calculated by summing the total weight (grams) of all HFSS products sold per store/day across each retailer.

Secondary outcomes include percentage point change in total HFSS sales by unit volume; percentage point change in sales of HFSS Specified Foods by weight and volume; repeated with stratification by HFSS category and percentage change in total calorie sales (in absolute and relative, per transaction terms).

#### Q2—What happened to retailer product portfolios after the introduction of the policy?

Changes to the product offering in-store are anticipated, due to reformulation, new product development and delisting. To assess whether significant product portfolio changes occurred following the legislation preimplementation and postimplementation product data cuts will be compared descriptively and via χ^2^ difference tests.

The hypothesis that there will be fewer HFSS products (in absolute and relative terms) available on the market following the legislation will be examined via the primary outcome; change in number (and proportion) of total HFSS products (vs non-HFSS). Secondary outcomes include change in number (and proportion) of Specified Foods; repeated with stratification by HFSS category.

Additionally, the hypothesis that reformulation and new product innovation will have led to healthier product portfolios postlegislation will be tested using paired t-tests or non-parametric equivalents. Outcomes include overall and category-level change in average NPM score time; change in A-point components: average energy, saturated fat, sugar and sodium per 100 g of product; change in C-point components: average protein and fibre/100 g and fruit, vegetable and nut percentage.

#### Q3—Has the HFSS legislation led to healthier overall purchasing using Eatwell Guide as metric?

Changes in sale-weighted product portfolios for each retailer against the Eatwell Guide segments (using the Wilcoxon signed-rank test) will be examined, with a focus on discretionary items, to understand if sales have shifted towards dietary recommendations following the legislation. Additionally, equitability will be explored by assessing differences in change to Eatwell Guide segment proportions by PPFI decile.

The primary outcome for this research question will be the difference in sales percentage (by weight) of discretionary items (pre vs postlegislation). Secondary analyses will assess; difference in sales percentage (by weight) in other Eatwell Guide segments; difference in percentage point change in discretionary product sales (by weight), stratified by PPFI decile; difference in sales percentage (by weight) in other Eatwell Guide segments, stratified by PPFI decile.

#### Q4—Were impacts of the HFSS legislation, determined by product sales (research question 1) and purchasing in line with the Eatwell guide (research question 3), equitable across different sociodemographic groups across the country?

Equitability of HFSS legislation impacts across different communities will be explored by repeating analysis for research question 1, with stratification by PPFI decile and the Index of Multiple Deprivation (IMD) decile and testing for effect heterogeneity across the PPFI and IMD groups.

### Data analysis plan

#### Data sources

Data will be provided for each retailer, covering three key data tables: sales data, product data, store data. Sales data will include daily store-level sales aggregates (weight, units and value (£)) for each product sold and will be provided by retailers for the whole study period (1 April 2021–30 September 2023), covering prelegislation and postlegislation.

Product data will include back of pack nutrition information (eg, nutrient values per 100 g of product), ingredients lists, product weight and HFSS legislation fields (HFSS category, NPM score, Specified Food status). Product data for retailer own brand products will be provided directly by retailers, and for branded products, this will be based on product data provided by NIQ Brandbank 2024. In addition, Brandbank will provide product information for retailer own brand products (where available) maximising coverage of both products and data fields. HFSS legislation fields will include supplier-entered data and calculated estimates (produced by retailers and/or Brandbank). Product data extracts will represent products available on the market at a minimum of four timepoints (1 October 2020, 1 October 2021, 1 October 2022 and 30 September 2023) spanning prelegislation and postlegislation.

Retailers will provide data on included stores, capturing location, store size (sales floor space), format and dates of legislation compliance roll out (where known). Additionally, open data, including the PPFI and IMD, will be spatially joined by store location to demographically describe store areas.

#### Data preprocessing

##### Data linkage

Data sources will be linked via unique product identifiers (daily sales and product data) and store identifiers (IDs) (daily sales and store data). Linkage with product data is important to identify HFSS products, so change in HFSS sales may be assessed. Match rates will be reported, indicating coverage of products with associated nutrition and HFSS information. Linkage with store data and associated area demographics are important for exploring change in HFSS sales by PPFI and IMD deciles.

##### High in salt, sugar or salt data

HFSS data fields were only required to be reported by suppliers to retailers at the point of legislation implementation (1 October 2022). Large rates of missingness in supplier-provided HFSS data fields are anticipated for prelegislation product data cuts. While Brandbank and retailers have produced calculated estimates for HFSS data fields, supplier-provided data are considered more reliable (due to suppliers having access to more complete product information via product specifications, eg, fruit, vegetable and nut (FVN) percentages) and shall be prioritised wherever possible. Where no HFSS data are available from supplier-entered data or Brandbank/retailer estimates, the Consumer Data Research Centre (CDRC) NPM calculator^29^ will be used to impute estimates based on available back of pack product information.

Exploratory data analysis (EDA) will reveal outliers and data quality issues. For example, supplier-inputted HFSS information is subjected to human input error, some of which may be systematic. Anecdotally, suppliers may enter zero values for HFSS data fields for products considered to be out of scope for the legislation (eg, it is not prepackaged or does not fall within one of the 13 legislation categories), these would indicate that the product NPM score was not assessed, rather than representing a true zero on this domain. Efforts will be made to identify and correct data errors.

##### Nutrient data

Back of pack nutrient values (presented per 100 g of product) are required for the calculation of NPM scores (and subsequent HFSS and ‘specified food’ categorisation), as well as for assessing total calorie sales. Therefore, completeness of nutrient values is also important and will be assessed via EDA. Nutrient information is often missing for products which are not sold prepackaged, such as fruits and vegetables, in-store bakery items and deli-counter goods. Where nutrient values are missing, UK Composition of Food Integrated Dataset^30^ food tables will be searched for the closest matching food item and used for imputation.

##### Product weight data

Product weights are required to allocate product sales by weight to segments of the Eatwell Guide and to calculate total calorie sales (calories sold=calories/100 g×total weight of product, summed across all products sold). Product weight information is requested as part of product data cuts provided by retailers and Brandbank but is often missing for products which are sold loose (eg, produce and in-store bakery). The CDRC has compiled ShelfScale^27^ an in-development dataset of generic product weights representing typical values for food items as sold rather than as eaten (eg, the weight of a whole pineapple). ShelfScale uses open data sources including the FSA’s Portion Size Handbook^31^ and UK Government analysis reports^32^ and will be used for weight imputation where close matches are available.

##### Eatwell Guide categorisation

Products will be mapped against the UK’s Eatwell Guide segments allowing sales to be expressed as a proportion of total purchases in each segment (by weight). The CDRC’s Eatwell Guide algorithm^33^ enables semiautomated classification of products to an extended list of Eatwell Guide segments based on product name and category. This semiautomation will be supplemented with coded quality assurance checks and a sample of manual validation.

### Data analysis

#### Data analysis environment

Analysis will take place in the Leeds Analytic Secure Environment for Research,^34^ a secure cloud-based trusted research environment (TRE). TREs are air locked and do not enable internet access, preserving commercially sensitive information. Each retailer’s data will be held in a separate TRE and aggregated outputs will be reported.

#### Analytical methods

**Figure 5:**
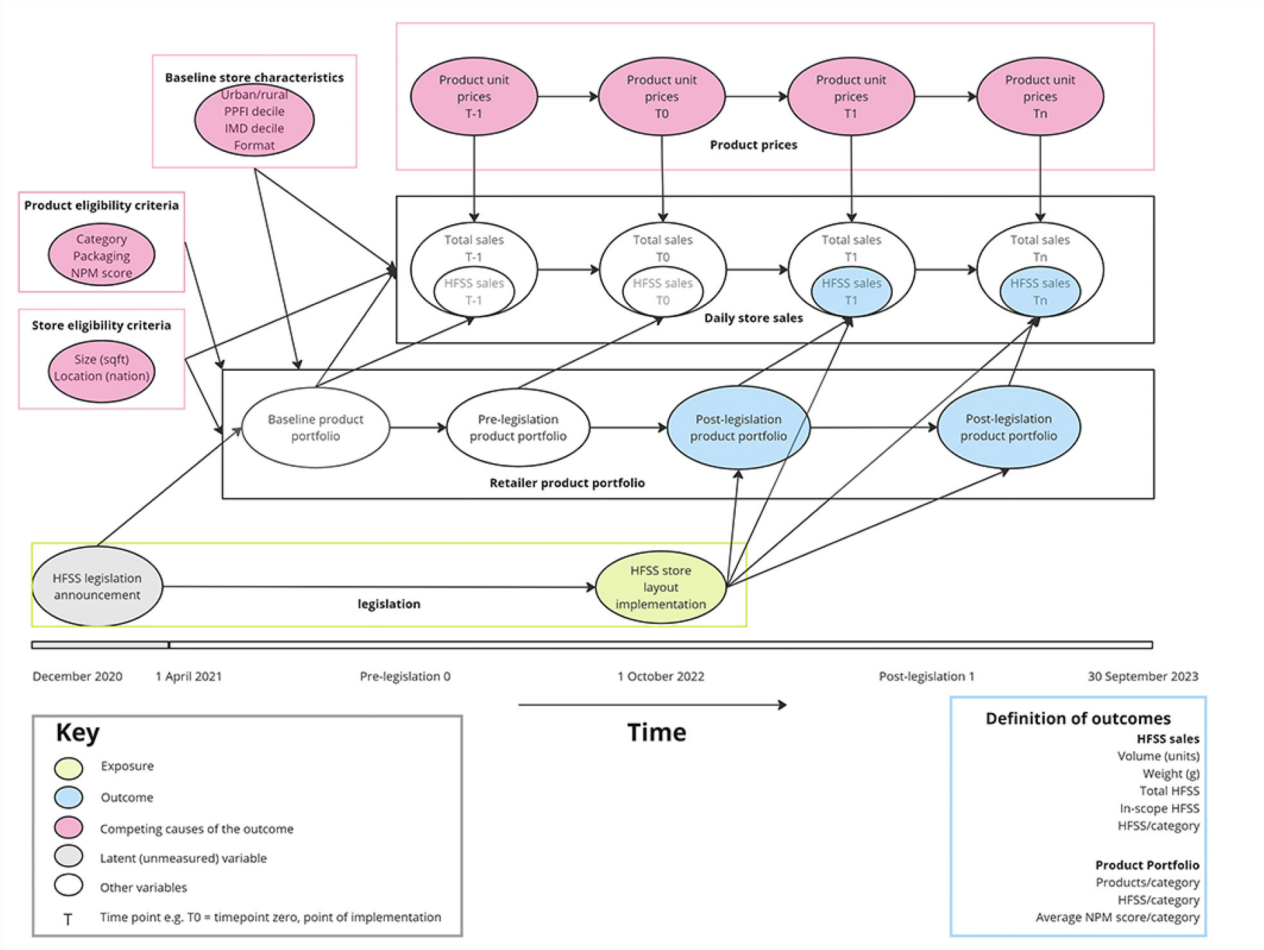
Directed acyclic graph (DAG) depicting variable selection for controlled interrupted time-series model to understand changes in HFSS sales. HFSS, high in saturated fat, sugar and/or salt; IMD, Index of Multiple Deprivation; NPM, Nutrient Profiling Model; PPFI, Priority Places for Food Index.

A controlled interrupted time-series ARIMA model, will be used to understand changes in sales of HFSS products postlegislation (research questions 1 and 3). Model covariates are depicted in figure 5. Research question 2, understanding changes in product portfolios will be addressed by descriptive statistics, χ^2^ tests (ie, for proportion of HFSS products by category) and group-wise paired difference tests (eg, t-test or non-parametric equivalent to assess differences in category-level NPM scores over time). Changes in the proportions of Eatwell categories by weight (as a proportion of total sales) will be assessed by the Wilcoxon signed rank test.

## Ethics and dissemination

This study has received approval from the Business, Environment and Social Sciences ethical review board at the University of Leeds (AREA 21–063).

Findings at the retailer and cross-retailer levels will be published in academic journal articles as well as industry-facing reports coproduced by IGD. Through meetings and workshops, we will disseminate results to inform future business practice and policymaking across the UK Devolved Nations.

## Data Availability

No data are available.
